# Lysophosphatidic Acid Acyltransferase β (LPAATβ) Promotes the Tumor Growth of Human Osteosarcoma

**DOI:** 10.1371/journal.pone.0014182

**Published:** 2010-12-01

**Authors:** Farbod Rastegar, Jian-Li Gao, Deana Shenaq, Qing Luo, Qiong Shi, Stephanie H. Kim, Wei Jiang, Eric R. Wagner, Enyi Huang, Yanhong Gao, Jikun Shen, Ke Yang, Bai-Cheng He, Liang Chen, Guo-Wei Zuo, Jinyong Luo, Xiaoji Luo, Yang Bi, Xing Liu, Mi Li, Ning Hu, Linyuan Wang, Gaurav Luther, Hue H. Luu, Rex C. Haydon, Tong-Chuan He

**Affiliations:** 1 Molecular Oncology Laboratory, Department of Surgery, The University of Chicago Medical Center, Chicago, Illinois, United States of America; 2 School of Pharmacy, Zhejiang University, Hangzhou, China; 3 Stem Cell Biology and Therapy Laboratory, The Children's Hospital of Chongqing Medical University, Chongqing, China; 4 Key Laboratory of Diagnostic Medicine designated by Chinese Ministry of Education, and Affiliated Hospitals of Chongqing Medical University, Chongqing, China; 5 School of Bioengineering, Chongqing University, Chongqing, China; 6 Department of Geriatrics, Xinhua Hospital of Shanghai Jiatong University, Shanghai, China; 7 Department of Cell Biology, Third Military Medical University, Chongqing, China; Indiana University, United States of America

## Abstract

**Background:**

Osteosarcoma is the most common primary malignancy of bone with poorly characterized molecular pathways important in its pathogenesis. Increasing evidence indicates that elevated lipid biosynthesis is a characteristic feature of cancer. We sought to investigate the role of lysophosphatidic acid acyltransferase β (LPAATβ, aka, AGPAT2) in regulating the proliferation and growth of human osteosarcoma cells. LPAATβ can generate phosphatidic acid, which plays a key role in lipid biosynthesis as well as in cell proliferation and survival. Although elevated expression of LPAATβ has been reported in several types of human tumors, the role of LPAATβ in osteosarcoma progression has yet to be elucidated.

**Methodology/Principal Findings:**

Endogenous expression of LPAATβ in osteosarcoma cell lines is analyzed by using semi-quantitative PCR and immunohistochemical staining. Adenovirus-mediated overexpression of LPAATβ and silencing LPAATβ expression is employed to determine the effect of LPAATβ on osteosarcoma cell proliferation and migration *in vitro* and osteosarcoma tumor growth *in vivo*. We have found that expression of LPAATβ is readily detected in 8 of the 10 analyzed human osteosarcoma lines. Exogenous expression of LPAATβ promotes osteosarcoma cell proliferation and migration, while silencing LPAATβ expression inhibits these cellular characteristics. We further demonstrate that exogenous expression of LPAATβ effectively promotes tumor growth, while knockdown of LPAATβ expression inhibits tumor growth in an orthotopic xenograft model of human osteosarcoma.

**Conclusions/Significance:**

Our results strongly suggest that LPAATβ expression may be associated with the aggressive phenotypes of human osteosarcoma and that LPAATβ may play an important role in regulating osteosarcoma cell proliferation and tumor growth. Thus, targeting LPAATβ may be exploited as a novel therapeutic strategy for the clinical management of osteosarcoma. This is especially attractive given the availability of selective pharmacological inhibitors.

## Introduction

Osteosarcoma (OS) is the most common primary malignancy of bone and accounts for ∼5% of childhood tumors in the United States with incidence peaking during the second decade of life [1]–[5]. The molecular pathogenesis underlying OS development remains to be thoroughly elucidated [4], [6]. At presentation, approximately 80% of patients are afflicted by some degrees of metastasis mandating management through chemotherapy and surgical resection [7]–[10]. Pulmonary metastasis remains the main cause of death in patients with OS [4], [11]. Many variants of OS are relatively resistant to current chemotherapy regimens [5], [12], [13]. We and others have reported that numerous genetic alterations may be found in OS tumors [4]–[6], [14]–[16]. However, it remains challenging to identify common genetic alterations that lead to OS development given the diversity and complexity in its pathogenesis [4], [17]–[21].

Lysophosphatidic acid acyltransferase (LPAAT, aka, 1-acylglycerol-3-phosphate O-acyltransferase 2, Agpat2) comprises a family of trans-membrane proteins consisting of at least six isoforms. The biological role of LPAAT is to convert lysophosphotidic acid (LPA) into phosphatidic acid (PA) [22], while only the α and β isoforms have significant acyltransferase activity [23]. LPAATβ expression is specific to heart, liver, adipose and pancreas [24]–[26]. Inherited mutation of LPAATβ is associated with lipodystrophy type 1, an autosomal recessive condition characterized by impaired triglyceride synthesis, low body fat percentage and insulin resistance [27].

PA is an important metabolite involved in phospholipid biosynthesis and membrane remodeling [28]. PA is considered an important secondary messenger capable of modulating pathways responsible for cell survival and proliferation, such as the mTOR and Raf-1 signaling cascade [29]–[32]. Increasing evidence indicates that enhanced lipid biosynthesis is a characteristic feature of cancer and deregulated lipogenesis plays an important role in tumor cell survival [33]–[36]. In fact, the oncogenic nature of lipogenesis closely depends on the activity and/or expression of key cancer-related oncogenes, such as HER2 [34]. Targeted gene deletion of *LPAATβ* in mice results in a complete absence of both white and brown adipose tissue [37], providing a biochemical link between the triglyceride synthesis pathway and adipogenesis in the liver and adipose tissue. Thus, it is conceivable that LPAATβ may play an important role in regulating cancer-related lipid metabolism.

We sought to investigate the role of LPAATβ in regulating OS cell proliferation and tumor growth. LPAATβ expression is readily detected in most OS cell lines. Exogenous expression of LPAATβ promotes OS cell proliferation and migration, while silencing LPAATβ expression inhibits cell proliferation and migration. Using an orthotopic xenograft model of human OS, we have demonstrated that exogenous expression of LPAATβ effectively promotes OS tumor growth, while knockdown of LPAATβ reduces OS tumor volume in the xenograft model. Taken together, our results strongly suggest that LPAATβ expression may be associated with aggressive phenotypes of human OS and that LPAATβ may play an important role in regulating OS cell proliferation and tumor growth. Therefore, targeting LPAATβ may be exploited as a novel therapeutic strategy for the clinical management of OS.

## Materials and Methods

### Tumor lines, cell culture and chemicals

No human subjects were used in the reported studies. However, tumor cell lines derived from patients were used, which has been approved by the Institutional Review Board (protocol #17043B). HEK293 and human OS lines 143B, SaOS2, MG63 and TE85 were purchased from ATCC (Manassas, VA). MG63.2 cell line was established by serially passaging the parental MG63 cells in mice followed by extraction and culture of pulmonary metastasis as previously described [38], [39]. Primary OS cells were isolated from resected OS specimens as previously described [15], [40], [41]. Cells were maintained in complete Dulbecco's Modified Eagle's Medium (DMEM) containing 10% FCS (fetal calf serum, HyClone, Logan, UT) at 37°C in 5% CO2. Unless otherwise indicated, all chemicals were purchased from Sigma-Aldrich (St Louis, MO) or Fisher Scientific (Pittsburgh, PA).

### Generation of recombinant adenoviruses expressing RFP, LPAATβ and siLPAATβ

Recombinant adenoviruses were generated using AdEasy technology as previously described [42]–[46]. Briefly, the coding region of mouse LPAATβ was PCR amplified and cloned into the adenoviral shuttle vector pAdTrace-TO4 and subsequently used to generate recombinant adenovirus in HEK293 cells. Similarly, four short interfering RNA (siRNA)-coding oligonucleotides against human LPAATβ were designed by using Dharmacon's *siDESIGN* program. The oligonucleotide cassettes were cloned into pSES shuttle vector as previously described [47]–[49]. All constructs were verified by DNA sequencing. Recombinant adenoviruses, designated as AdR-LPAATβ and AdR-siLPAATβ, were generated as previously described [42]–[46]. AdR-LPAATβ and AdR-siLPAATβ express RFP as a marker for monitoring infection efficiency. Analogous adenovirus expressing monomeric RFP (Ad-RFP) was used as control [44], [50], [51].

### RNA isolation and semi-quantitative RT-PCR analysis

Total RNA was isolated using TRIZOL Reagents (Invitrogen) and cDNA was generated via reverse transcription reaction with hexamer and Superscript II RT (Invitrogen). The first strand cDNA products were further diluted 5- to 10-fold and used as PCR templates. Semi-quantitative RT-PCR was carried out using PCR primers designed by using the Primer3 program to amplify the gene of interest (approximately 150–180 bps). Primers used were as follows: human LPAATβ, 5′- CCT TCC TCC ACA TCT CCA AG-3′ and 5′- CCG GAC AGA GTG GTA TTT GG-3′; human GAPDH, 5′- CAA CGA ATT TGG CTA CAG CA-3′ and 5′- AGG GGA GAT TCA GTG TGG TG-3′; and mouse LPAATβ, 5′-GCG GAC AGA AGA AAC TGG AG-3′ and 5′-TGA AGT AGA CAC CCC CAA GG-3′. Touchdown PCR reactions were carried out as previously described [15], [41], [48], [49], [52]–[54]. PCR products were separated via 1.2% agarose gels. The resulting bands were analyzed in Kodak ImageStation 440CF using Kodak 1D 3.6 software.

### Crystal violet staining

Subconfluent cells were seeded in 12-well plates and infected with an optimal titer of AdR-LPAATβ, AdR-siLPAATβ or AdRFP. Cells were subjected to crystal violet staining at 5 days after infection as previously described [41], [55]. Images were taken from the plates relative staining intensities were analyzed by using ImageJ software.

### Cell proliferation (MTT) assay

MTT assay was used to quantitatively analyze cell proliferation as previously described [15], [41], [56]. Briefly, cells were seeded in 96-well plates and infected with AdR-LPAATβ, AdR-siLPAATβ or AdRFP. Cell proliferation was assessed by MTT assay daily for five days. Optical absorbance was measured at 570 nm using a 96-well microplate reader. All experiments were performed in triplicate.

### Cell migration and wound healing assay

The experiments were carried out essentially as described previously [15], [16], [39], [41], [53], [57]. Subconfluent cells were plated into 6-well plates and infected with AdR-LPAATβ, AdR-siLPAATβ or AdRFP in 1% FCS containing medium. At 16 h after infection, the monolayer of cells was wounded using micropipet tips. Marks were created on the plates using 18-gauge needles as reference points for serial imaging. Bright field images of the same field were taken at 4, 12, 24, and 36 hours. The results were repeated in at least two batches of experiments.

### Orthotopic tumor model of human osteosarcoma

The reported animal work was carried out according to the guidelines approved by the Institutional animal Care and Use Committee (Protocol #71328). Athymic nude (nu/nu) mice (4–6 week-old, male) were used (5 mice per group, Harlan Laboratories, Indianapolis, IN). Human OS cells (e.g., 143B or MG63) were stably transduced with a retroviral vector expressing firefly luciferase (namely 143B-Luc or MG63-Luc). Cells were seeded in 100 mm cell culture dishes and infected with AdR-LPAATβ, AdR-siLPAATβ or AdRFP. At 36–48 h after infection cells were collected and resuspended in PBS to a final concentration of 2×10^7^ cells/ml. 50 ul of the resuspened cells (i.e., 10^6^ cells/injection) were periosteally injected posterior to the proximal tibia using a 25 gauge needle.

### Xenogen bioluminescence imaging

Xenogen imaging was carried out as described [15], [41]. Animals were anesthetized with isoflurane. Approximately 10 min prior to using Xenogen IVIS 200 imaging system, animals were injected (ip) with D-Luciferin sodium salt (Gold BioTechnology) at 100 mg/kg in 0.1 ml sterile PBS. The pseudoimages were obtained by superimposing the emitted light over the gray-scale photographs of the animal. Quantitative analysis was done with Xenogen's Living Image V2.50.1 software.

### Tumor volume measurement

The dimensions of the primary tumor sites were measured every 3–4 days. The volume was calculated as previously reported [38], [39], by using the following equation: volume  =  (L+W)(L)(W)(0.2618). The width (W) was an average of the distance at the proximal tibia at the level of the knee joint in the anterior-posterior and medial-lateral planes. The length (L) was the distance from the most distal extent of the calf musculature or distal tumor margin to the knee joint or proximal tumor margin [39].

### Histological analysis

Animals were sacrificed and the primary tumors were harvested at necropsy and fixed in 10% formalin or decalcification solution (Cal-Ex, Fisher Scientific). The fixed samples were embedded in paraffin. Sections were stained with hematoxylin and eosin, and analyzed under a microscope.

### Immunohistochemical staining

Immunohistochemical analysis was carried out as previously described previously [14]–[16], [39], [41]. Briefly, paraffin sections were deparaffinized, rehydrated and probed with anti-LPAATβ, anti-PCNA, or isotype IgG, followed by incubation with secondary antibodies conjugated with HRP. The expression of expected proteins was visualized by DAB staining and examined under a microscope. Stains without the primary antibody, or with control IgG, were used as negative controls.

### Statistical analysis

Microsoft Excel was used to calculate standard deviations (SD) and statistically significant differences between samples using the two-tailed Student's t-test. For all quantitative assays, each assay condition was performed in triplicate and the results were repeated in at least three independent experiments. All collected data were subjected to statistical analysis. A *p-value <0.05* was defined as statistically significance.

## Results

### LPAATβ is expressed in most human OS cells

**Figure 1:**
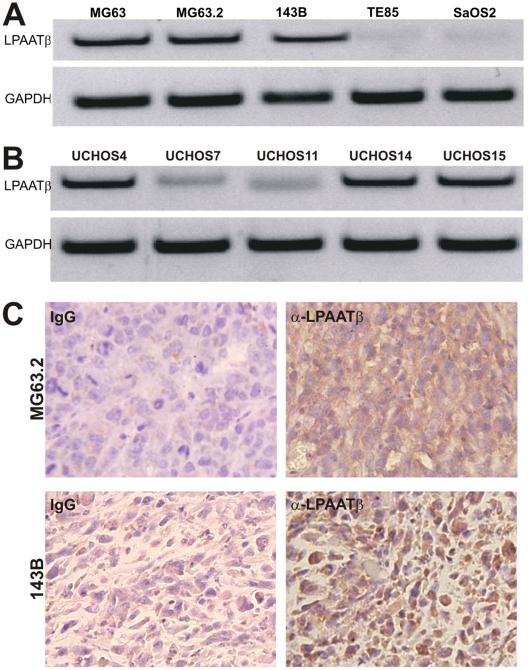
Endogenous expression of LPAATβ in human osteosarcoma cell lines. **A**. Semi-quantitative RT-PCR analysis of LPAATβ expression in the five commonly used OS lines MG63, MG63.2, 143B, TE85, and SaOS2. GAPDH expression was used as an internal control. **B**. Semi-quantitative RT-PCR analysis of LPAATβ expression in the five primary OS cell lines derived from OS tumors. GAPDH expression was used as an internal control. **C**. Immunohistochemical staining of LPAATβ expression in xenograft tumors formed by MG63.2 and 143B cells in athymic nude mice. Expression of LPAATβ was detected by using a LPAATβ antibody. Isotype IgG was used as a control for immunohistochemical staining.

In order to understand the possible role of LPAATβ in human OS tumorigenesis and progression, we analyzed the endogenous expression of LPAATβ in a panel of human OS cell lines. We first assessed the expression of LPAATβ in the four commercially available OS lines (i.e., MG63, 143B, TE85, and SaOS2) and the line MG63.2 derived from MG63 [38]. As shown in **Fig. 1A**, expression of LPAATβ was readily detected in three of the five lines. Interestingly, expression of LPAATβ is more apparent in lines with higher xenogenic tumor growth potential, such as 143B, MG 63, and MG63.2 as compared to lines with less tumorigenic capacity, such as TE85 and SaOS2 [39]. We next examined the expression of LPAATβ in five OS lines derived from OS patients [15], [40], [41]. Three of the five patient-derived OS lines exhibited apparent expression of LPAATβ while other two lines showed weak but detectable expression of LPAATβ (**Fig. 1B**). Our previous studies indicated that UCHOS4 and UCHOS15 lines were more tumorigenic than UCHOS11 cells [15], [40], [41]. The expression of LPAATβ at protein level was further confirmed in xenograft tumors formed by 143B and MG63.2 cells (**Fig. 1C**). Taken together, these results indicate that LPAATβ is commonly expressed in human OS cells and that LPAATβ expression is seemingly correlated with aggressive phenotype of OS cell lines'.

### LPAATβ promotes cell viability and proliferative activity of OS cells

**Figure 2:**
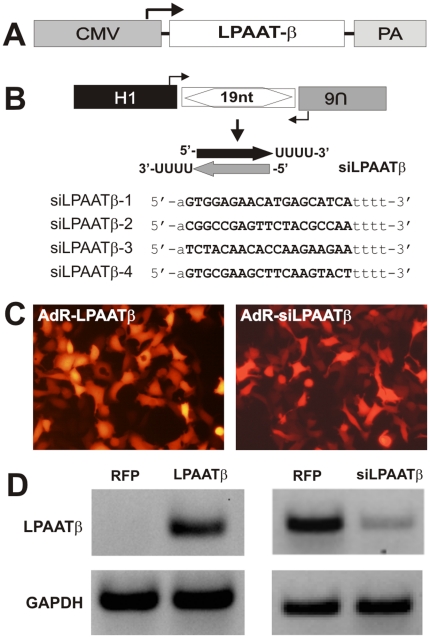
Construction and characterization of recombinant adenovirus that expresses mouse LPAATβ or knocks down human LPAATβ. **A**. Schematic representation of adenoviral shuttle vector that overexpresses LPAATβ driven by CMV promoter (for making AdR-LPAATβ). The shuttle vector also expresses RFP marker. **B**. Schematic representation of adenoviral pSOS shuttle vector that expresses siRNA driven by dual opposing promoters (for making AdR-siLPAATβ). The pSOS shuttle vector also expresses RFP marker. The four siRNA targeting sites against human LPAATβ are listed. **C**. Adenovirus-mediated effective transduction of human osteosarcoma cells. Optimal titer of the recombinant adenoviruses AdR-LPAATβ and AdR-siLPAATβ were used to infect 143B cells. The expression of red fluorescent protein (RFP) was examined at 48 h after infection. **D**. Characterization of AdR-LPAATβ-mediated overxpression and AdR-siLPAATβ-mediated knockdown. Subconfluent 143B cells were infected with AdR-LPAATβ, AdR-siLPAATβ, or AdRFP control for 48 h. Total RNA were collected and subjected to RT-PCR. The resultant cDNA was subjected to semi-quantitative PCR using primers specific for mouse LPAATβ mRNA (for AdR-LPAATβ infection) or human LPAATβ mRNA (for AdR-LPAATβ infection). GAPDH was used as an internal control to normalize all samples.

In order to further investigate the functional role of LPAATβ in OS proliferation and tumor growth, we sought to generate recombinant adenoviruses that can effectively over-express LPAATβ (AdR-LPAATβ) and siRNAs targeting human LPAATβ (AdR-siLPAATβ) using the AdEasy technology [44], [45]. We constructed an adenoviral vector that over-expresses mouse LPAATβ driven by a CMV promoter (**Fig. 2A**). The siRNA expressing adenoviral vectors were constructed using the pSOS system [46], [47], [49], for which four target sites were chosen (**Fig. 2B**). The generated adenoviruses, which also express the RFP maker, were shown to effectively transduce 143B cells (**Fig. 2C**) and other OS lines (data not shown). We further demonstrated that AdR-LPAATβ effectively overexpressed mouse LPAATβ mRNA while human LPAATβ mRNA was significantly knocked down by AdR-siLPAATβ (**Fig. 2D**), indicating that the adenoviral vectors are functional.

**Figure 3:**
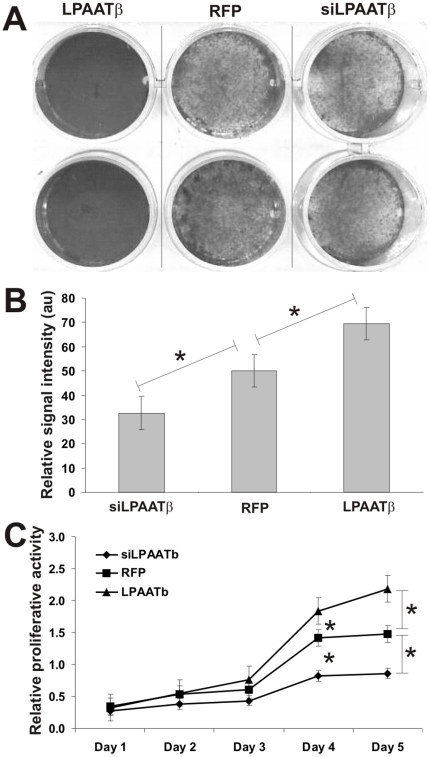
Effect of LPAATβ on osteosarcoma cell proliferation. **A**. Crystal violet staining assay for cell viability. 143B cells were seeded in 12-well plates and infected with an optimal titer of AdR-LPAATβ, AdR-LPAATβ, or AdRFP control. Viable cells were subjected to crystal violet staining at 5 days after infection. Representative duplicate staining is shown. **B**. Quantitative analysis of the crystal violent staining assay. Relative staining intensities were measured by using ImageJ software. “*” *p-value <0.05*. **C**. MTT cell proliferation. 143B cells were seeded in 96-well plates and infected with an optimal titer of AdR-LPAATβ, AdR-LPAATβ, or AdRFP control (in triplicate). At the indicated time points after infection, cells were subjected to MTT assay to determine relative proliferative activity. “*” *p-value <0.01*.

Using these adenoviral vectors, we investigated the effect of overexpression or knockdown of LPAATβ on OS cell viability and proliferation. Using the crystal violet viability assay, we found that over-expression of LPAATβ promoted 143B proliferation and increased viable cells, whereas knockdown of LPAATβ led to a decrease in cell proliferation and viability of 143B cells (**Fig. 3A**). Quantitative analysis of the crystal violet staining results indicated that the increase in cell viability and proliferation resulted from LPAATβ overexpression or the decrease in cell viability and proliferation caused by LPAATβ knockdown was statistically significant when compared with the control cells (**Fig. 3B**). The effect of LPAATβ on OS cell proliferation was further assessed by MTT assay. Consistent with the results obtained from crystal violet staining assay, overexpression of LPAATβ promoted OS cell proliferation while silencing LPAATβ expression inhibited cell proliferation (**Fig. 3C**). These results were reproducible in other OS cell lines (data not shown). Taken the above findings together, LPAATβ expression can enhance OS cell viability and proliferation *in vitro*.

### LPAATβ enhances OS cell migration in vitro

**Figure 4:**
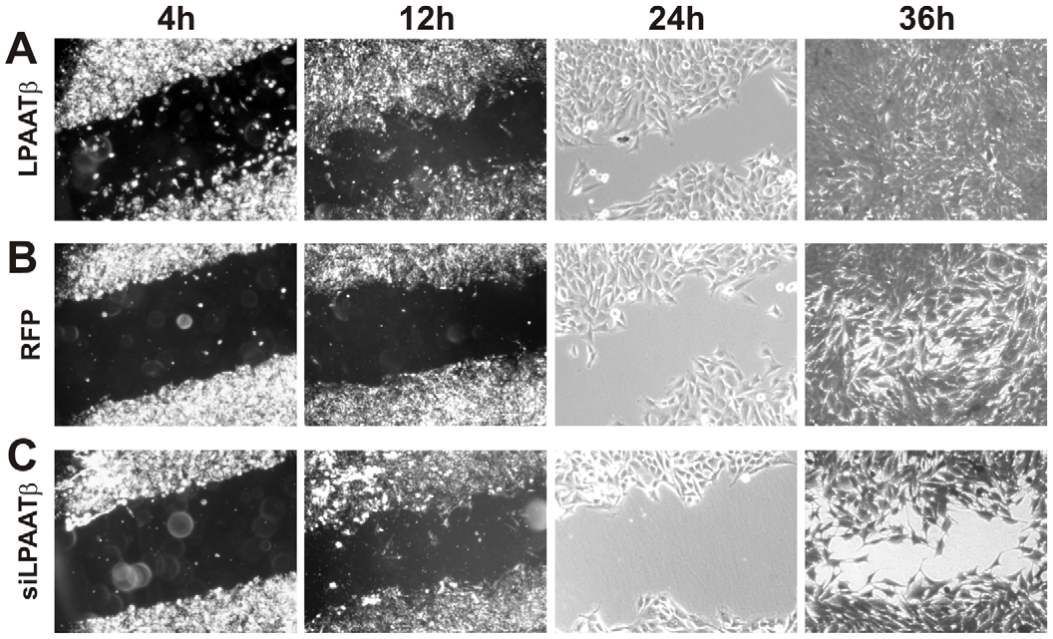
Effect of LPAATβ on osteosarcoma cell migration (wound healing assay). Subconfluent 143B cells were seeded in 6-well plates and infected with AdR-LPAATβ (**A**), AdRFP (**B**), or AdR-siLPAATβ (**C**) for 24 h in growth media containing 1% FCS. A scratch wound was introduced across the subconfluent monolayer of cells in each well. Bright field images of the same fields were recorded at the indicated time points. The assays were repeated in at least two independent batches of experiments. Representative results are shown.

The ability of tumor cells to migrate is considered an important indicator of tumor cell's aggressiveness. To assess the effect of LPAATβ on OS cell migration *in vitro*, we conducted the commonly-used cell wounding experiment [15], [16], [39], [41], [53], [57]. We found that over-expression of LPAATβ effectively promoted a faster wound healing than that of the control cells (**Fig. 4A** *vs.* **4B**). Conversely, knockdown of LPAATβ expression inhibited cell migration and wound healing, at least in the duration of the testing period (**Fig. 4C** *vs.* **4B**). Similar results were obtained from MG63.2 and other OS cell lines (data now shown). Thus, the above results strongly suggest that LPAATβ expression may be related to OS cell migration-related aggressiveness phenotype.

### Overexpression of LPAATβ promotes tumor growth in an orthotopic model of human OS tumors

**Figure 5:**
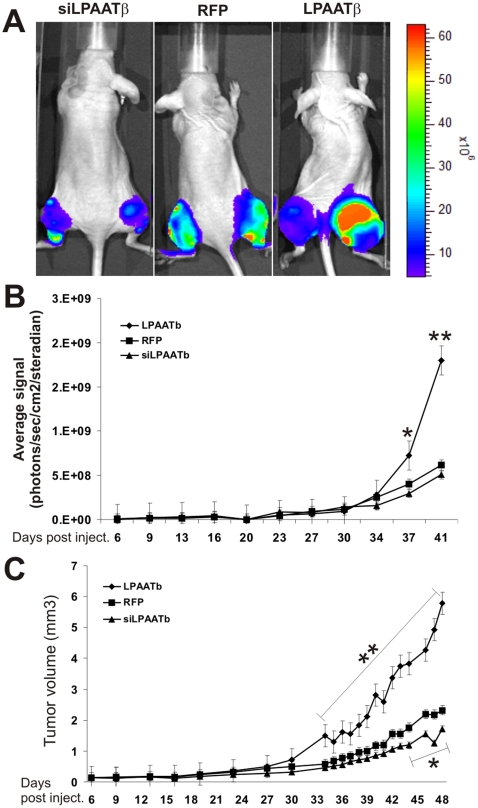
Effect of LPAATβ on tumor growth in the orthotopic model of osteosarcoma. **A**. Xenogen IVIS 200 bioluminescence imaging of orthotopic tumor growth. Athymic nude mice were periosteally injected at proximal tibia with 143B cells infected with AdR-LPAATβ, AdRFP, or AdR-siLPAATβ for 15 h (10^6^ cells/injection). Animals were subjected to Xenogen imaging at the indicated time points. Representative imaging results at 37 days post injection are shown. **B**. Quantitative analysis of Xenogen imaging signal intensity (photons/sec/cm^2^/steradian) over the time after injection. **C**. Tumor growth as determined by tumor volume. The dimensions of injection sites were measured roughly every 3 days during the course of study. Tumor volumes (mm^3^) were calculated as described in Methods. “*” *p-value <0.05*, “**” *p*-*value <0.001*.

We further analyzed the effect of LPAATβ expression on OS tumor growth *in vivo*. Using our previously established orthotopic tumor model of human OS [15], [38], [39], [41], we found that 143B cells transduced with LPAATβ formed much larger tumors than that of the control group's, whereas knockdown LPAATβ led to the inhibition of tumor growth (**Fig. 5A**). Quantitative analysis of the Xenogen bioluminescence imaging data revealed that overexpression of LPAATβ significantly promoted OS tumor growth, especially at the late stage (*p<0.05*) while knockdown of LPAATβ did not significantly affect Xenogen imaging signal (**Fig. 5B**).

Although Xenogen bioluminescence imaging is sensitive and quantitative, the imaging results may not be reliable when tumors have necrosis and/or are less vascularized. Thus, we also measured the tumor sizes during the course of the study, and found that the overall tumor growth trends were in general consistent with that of Xenogen imaging analysis (**Fig. 5C**). However, the tumor volume was reduced in the LPAATβ knockdown group (*p<0.05*). The calculated doubling time was 9.30 days for RFP control group, 11.99 days for the siLPAATβ group, and 6.64 days for the LPAATβ group, indicating that tumors with LPAATβ over-expression had a significantly shorter doubling time than that of the control group (*p<0.02*).

**Figure 6:**
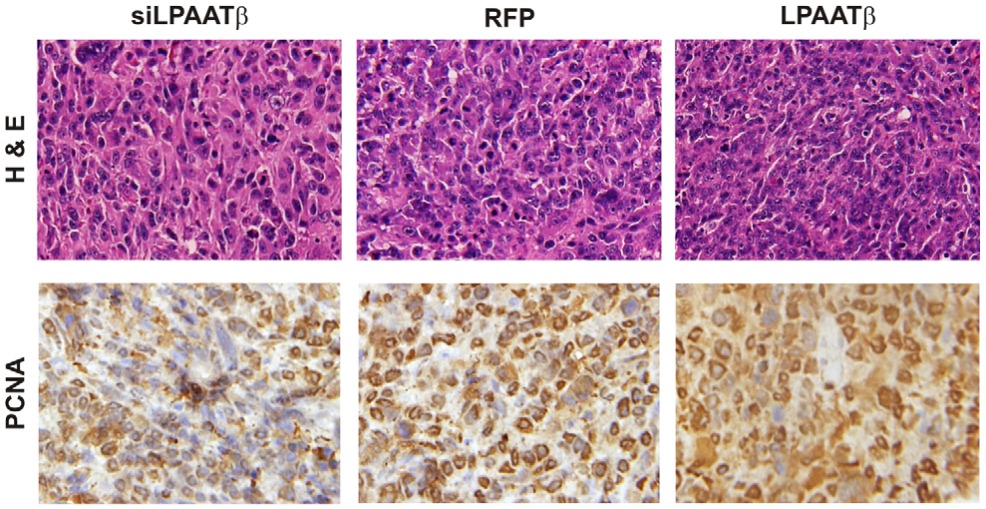
Histological evaluation of xenograft osteosarcoma tumors. Tumors were retrieved from animals and subjected to paraffin-embedded. Sections were subjected to Hemotoxylin & Eosin staining (top panel) and PCNA antibody immunohistochemical staining (bottom panel). Representative results are shown.

We further conducted histologic examination on the retrieved tumor samples. H & E staining revealed that the LPAATβ overexpression group exhibited increased cell numbers with relatively higher nuclear to cytoplasmic ratio (1.7), while the LPAATβ knockdown group has demonstrates relatively lower nuclear to cytoplasmic ratio (0.73) When compared to RFP group (1.2) (**Fig. 6** top panel). Proliferative activity of the tumor cells was assessed by anti-PCNA immunohistochemical staining. Consistent with H & E staining, the LPAATβ overexpression group exhibited increased cell proliferation whereas the LPAATβ knockdown group showed relatively low proliferative activity (**Fig. 6** bottom panel). Taken together, these *in vivo* results strongly suggest that LPAATβ may play an important role in promoting OS tumor growth.

## Discussion

OS is the most common primary malignancy of bone [1]–[4], [58]. Although clinical management of OS has significantly improved, survival rate in the last few decades has plateaued. This bottle-neck has been a consequence of our poor understanding of the molecular mechanism underlying OS development and progression. Here, we investigate the role of LPAATβ in OS cell proliferation and tumor growth. LPAATβ expression is readily detected in most OS cell lines. Exogenous expression of LPAATβ promotes OS cell proliferation and migration, while silencing LPAATβ expression inhibits cell proliferation and migration. Using an orthotopic xenograft model of human OS, we demonstrate that exogenous expression of LPAATβ effectively promotes OS tumor growth, while knockdown of LPAATβ reduces OS tumor volume in the xenograft model. These results suggest that LPAATβ may play an important role in regulating OS cell proliferation and tumor growth.

Our *in vitro* results suggest that the expression level of LPAATβ may correlate with OS malignant characteristics. We have found an increased level of LPAATβ in tumors with highly malignant OS lines, such as MG63.2, 143B and UCHOS4, whereas LPAATβ expression level is lower in OS lines that are more differentiated and with lower malignant characteristics, such as TE85 and SaOS [15], [39]. However, further studies on LPAATβ expression level needs to be carried out on a large set of clinical OS samples in order to determine if LPAATβ expression may serve as an indicator of OS aggressive phenotypes.

LPAATs are a family of enzyme that catalyses the biosynthesis of PA [22]. Overexpression of LPAATβ has been shown to transform cells *in vitro* [59]. LPAATβ is highly expressed in advanced ovarian tumors and is associated with aggressive histology and decreased overall survival [60]–[63]. Chemical inhibitors of LPAATβ exhibit anti-tumor activity and promote apoptosis in lymphomas, acute leukemia, and multiple myeloma [59], [64]–[67]. Inhibition by either genetic means or by isoform-specific small molecules results in a block to cell signaling pathways and apoptosis [59].

PA is a versatile lipid second-messenger that functions as a cofactor in several critical signaling pathways in cancer cells. For example, the full activation of c-Raf-1 and B-raf is manifested only when phosphatidic acid physically interacts with a polybasic amino acid segment in these kinases [28]. This interaction is required for the translocation of Raf kinases to the plasma membrane where they phosphorylate and activate their targets. Moreover, binding of phosphatidic acid to a polybasic domain in mTOR is essential for its activation [30]. Thus, the role of phosphatidic acid is central to the regulation of proteins in both proliferative and survival pathways in tumor cells. We found that silencing LPAATβ expression in osteosarcoma cells resulted in decreased expression of c-Myc, cyclin D1, c-Fos, and MDM2, but an increased Rb expression (data not shown). Nonetheless, the exact role of LPAATβ in OS development remains to be investigated.

It has been reported that inhibition of LPAATβ with small interfering RNA or selective inhibitors CT32521 and CT32228 induces apoptosis in human ovarian and endometrial cancer cell lines *in vitro* and enhances the survival of mice bearing ovarian tumor xenografts [61]. Previous studies have also demonstrated that inhibition of LPAATβ expression with siRNA in mammalian cells suppresses basal Erk phosphorylation [68]. Inhibition of LPAATβ with small-molecule antagonists prevents the translocation of Raf to the plasma membrane and subsequent Erk phosphorylation [68]. These inhibitors also suppress the activation of proteins in the phosphoinositide-3-kinase/Akt pathway, including Akt, mTOR, and S6 kinase [68]. Therefore, it is conceivable that LPAATβ may be exploited as a novel cancer drug target for OS clinical management.

Taking together, we have conducted *in vitro* and *in vivo* studies to investigate the role of LPAATβ in OS cell proliferation and tumor growth. Our results indicate that exogenous expression of LPAATβ promotes OS cell proliferation and migration, while silencing LPAATβ expression inhibits cell proliferation and migration. Furthermore, exogenous expression of LPAATβ effectively promotes OS tumor growth, while knockdown of LPAATβ reduces OS tumor volume in an orthotopic xenograft model of human OS. These results strongly suggest that LPAATβ expression may be associated with aggressive phenotypes of human OS and that LPAATβ may play an important role in regulating OS cell proliferation and tumor growth. Therefore, targeting LPAATβ may be exploited as a novel therapeutic strategy for OS clinical management. This is especially attractive given the availability of selective pharmacological inhibitors.
